# Supplementary material for: Exploring the Potential Mechanism of Action of Piperine against Candida albicans and Targeting Its Virulence Factors
Source: Biomolecules. 2023 Nov 30;13(12):1729. doi: 10.3390/biom13121729 (PMC10742119; doi:10.3390/biom13121729)
Supplement: Supplementary file 1 [file biomolecules-13-01729-s001.zip › biomolecules-2628383-supplementary.pdf]

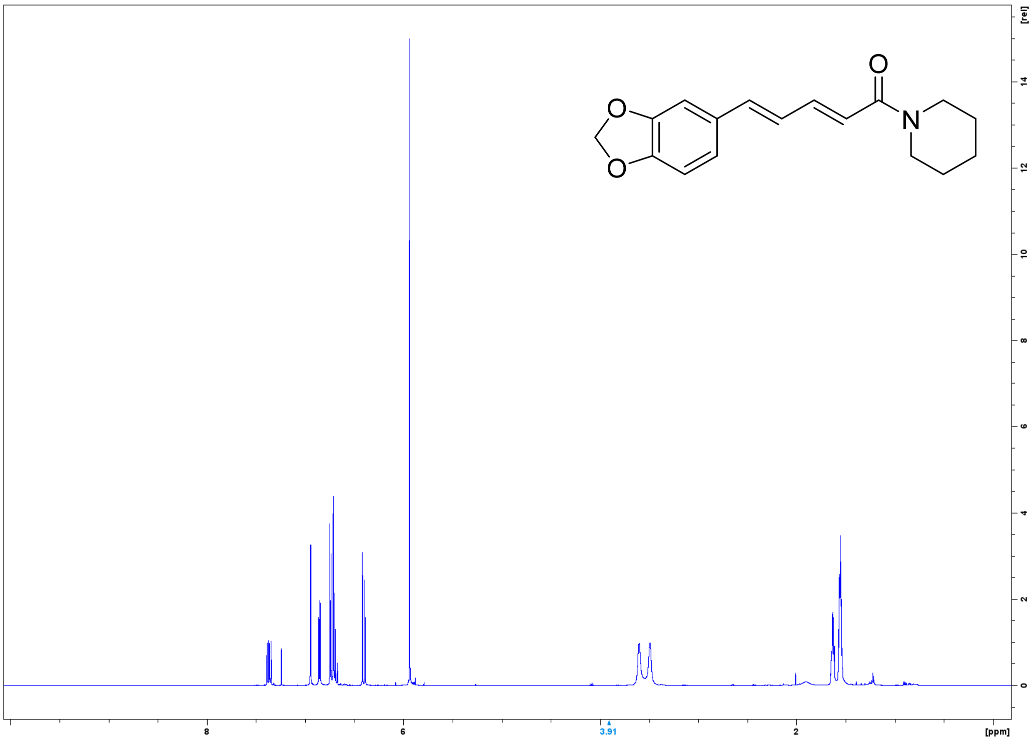

Figure S1. ^{1}H NMR spectrum of compound **1**.

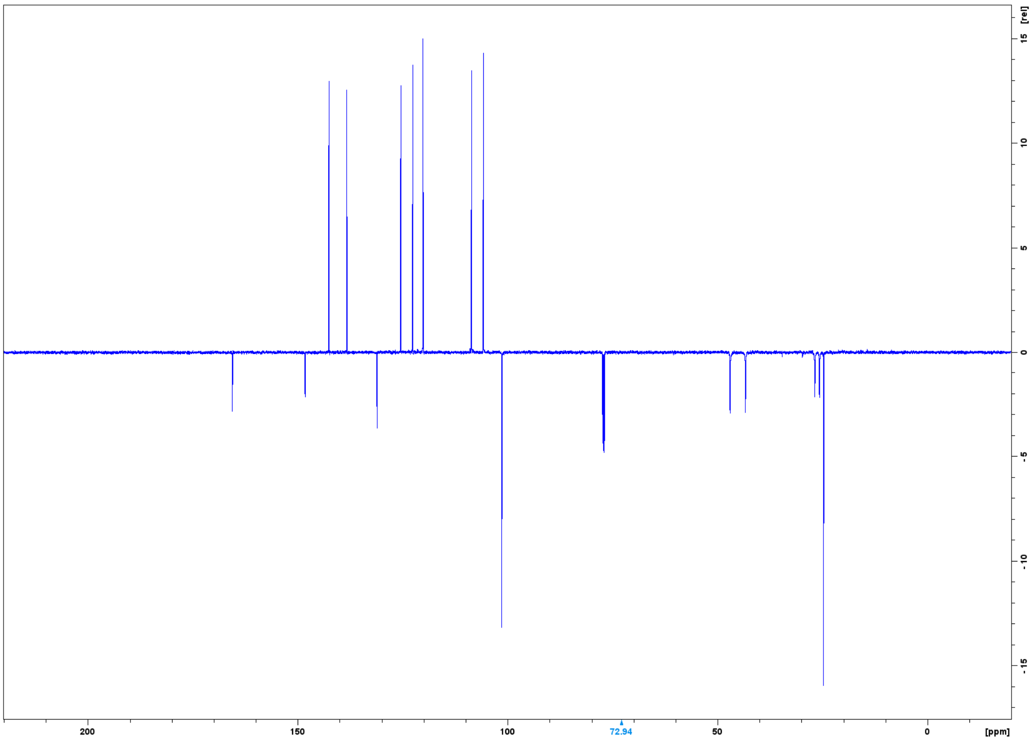

Figure S2. DEPTQ spectrum of compound **1**.

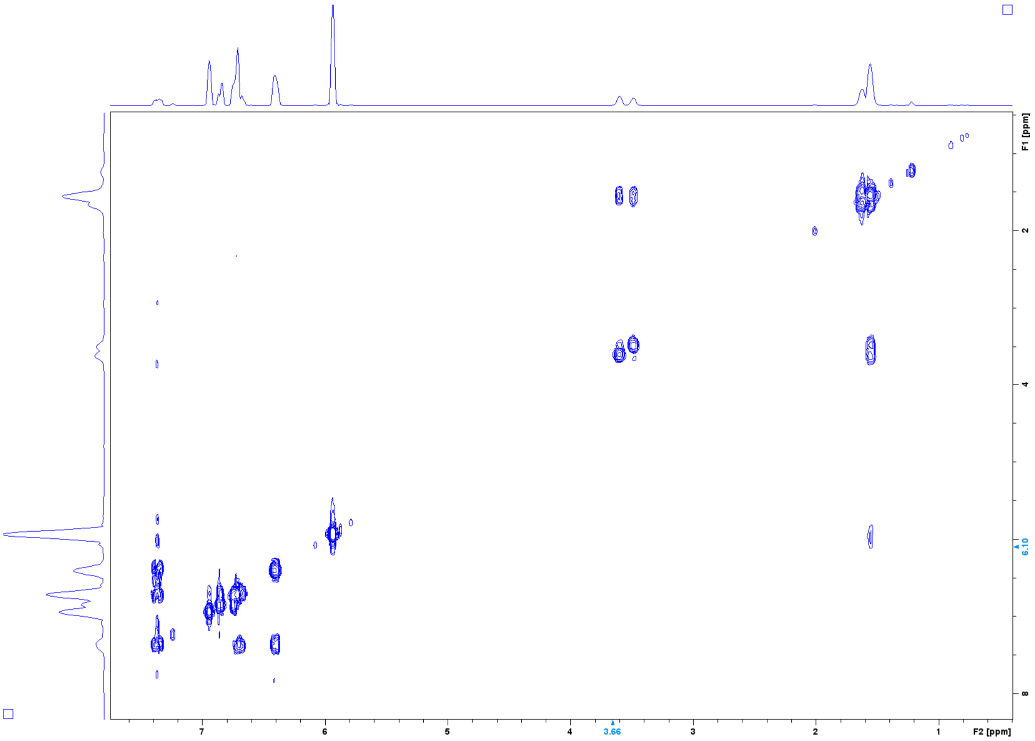

Figure S3. ^1H-^1H COSY spectrum of compound **1**.

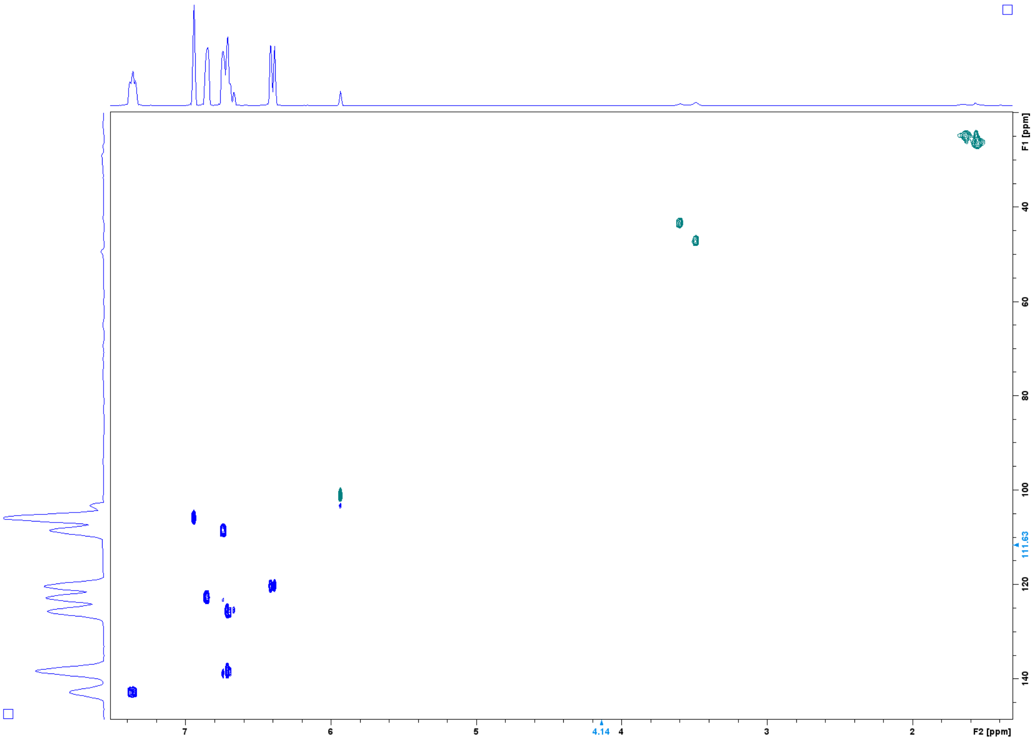

Figure S4. HSQC spectrum of compound **1**.

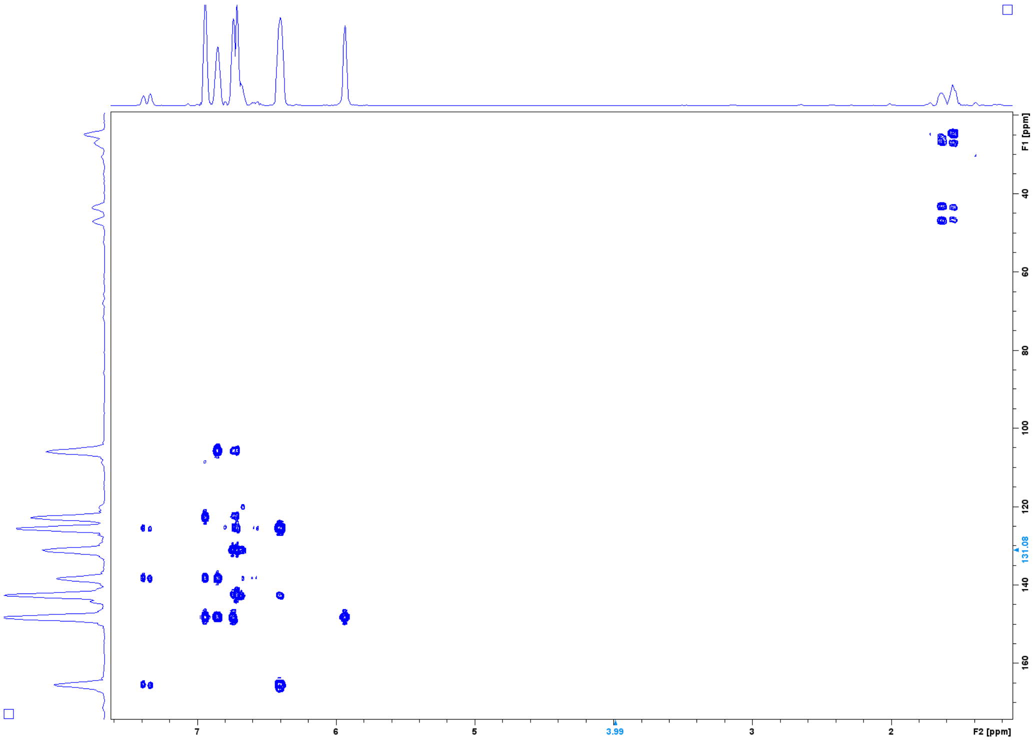

Figure S5. HMBC spectrum of compound **1**.

Table S1. List of heterozygous haploid deletion strains used in this study.

Strains*	Gene ontology (GO)**
C. albicans efg1Δ/EFG1	bHLH transcription factor; required for white-phase cell type, RPMI and Spider biofilm formation, hyphal growth, cell-wall gene regulation; roles in adhesion, virulence.
C. albicans cph2Δ/CPH2	Myc-bHLH transcription factor; promotes hyphal growth; directly regulates Tec1 to induce hypha-specific genes; probably homodimeric, phosphorylated; required for colonization of the mouse GI tract; rat catheter and Spider biofilm induced.
C. albicans hwp2Δ/HWP2	GPI-anchored, glycosylated cell wall protein; required for biofilm formation, adhesion, filamentous growth on some media; expressed in hyphae; mutant delayed in virulence; regulated by Efg1, Tup1; similar to Hwp1 and Rbt1 domains.
C. albicans ume6Δ/UME6	Zn(II)2Cys6 transcription factor; has a long 5'-UTR that regulates translational efficiency and controls transition to filamentous growth; stability controlled by Grr1p, Ubr1p, Ptc2p in response to CO_2 and O_2 levels.
C. albicans flo8Δ/FLO8	Transcription factor; required for hyphal formation and CO_2 induced white-opaque switching; activates ERG6 and regulates hyphal gene expression; required for virulence in mice; binds Efg1p and Mss1p.
C. albicans tup1Δ/TUP1	Transcriptional corepressor; represses filamentous growth; regulates switching; role in germ tube induction, farnesol response; in repression pathways with Nrg1, Rfg1; farnesol upregulated in biofilm; rat catheter, Spider biofilm repressed.
C. albicans nrg1Δ/NRG1	Transcription factor/repressor; regulates chlamydospore formation/hyphal gene induction/virulence and rescue/stress response genes; effects both Tup1 dependent and independent regulation; flow model biofilm induced; Spider biofilm repressed.
C. albicans ali1Δ/ALI1	Putative NADH-ubiquinone oxidoreductase; in detergent-resistant membrane fraction (possible lipid raft component); predicted N-terminal acetylation; nitric oxide-repressed; plasma membrane-localized; protein decreases in stationary phase
C. albicans cox4Δ/COX4	Putative cytochrome c oxidase subunit IV; Mig1-regulated; macrophage/pseudohyphal-induced gene; macrophage-induced protein; repressed by nitric oxide; 5'-UTR intron; Hap43-repressed.

C. albicans hog1Δ/HOG1	MAP kinase of osmotic-, heavy metal-, and core stress response; role in regulation of response to stress; phosphorylated in response to H_2O_2 or NaCl; acts as repressor of START; mutant induces protective mouse immune response.

*Source: Adapted with permission from *Candida albicans* GRACE (gene replacement and conditional expression) library [29]. **Skrzypek MS, Binkley J, Binkley G, Miyasato SR, Simison M, and Sherlock G. "Candida Genome Database" http://www.candidagenomedatabase.org/

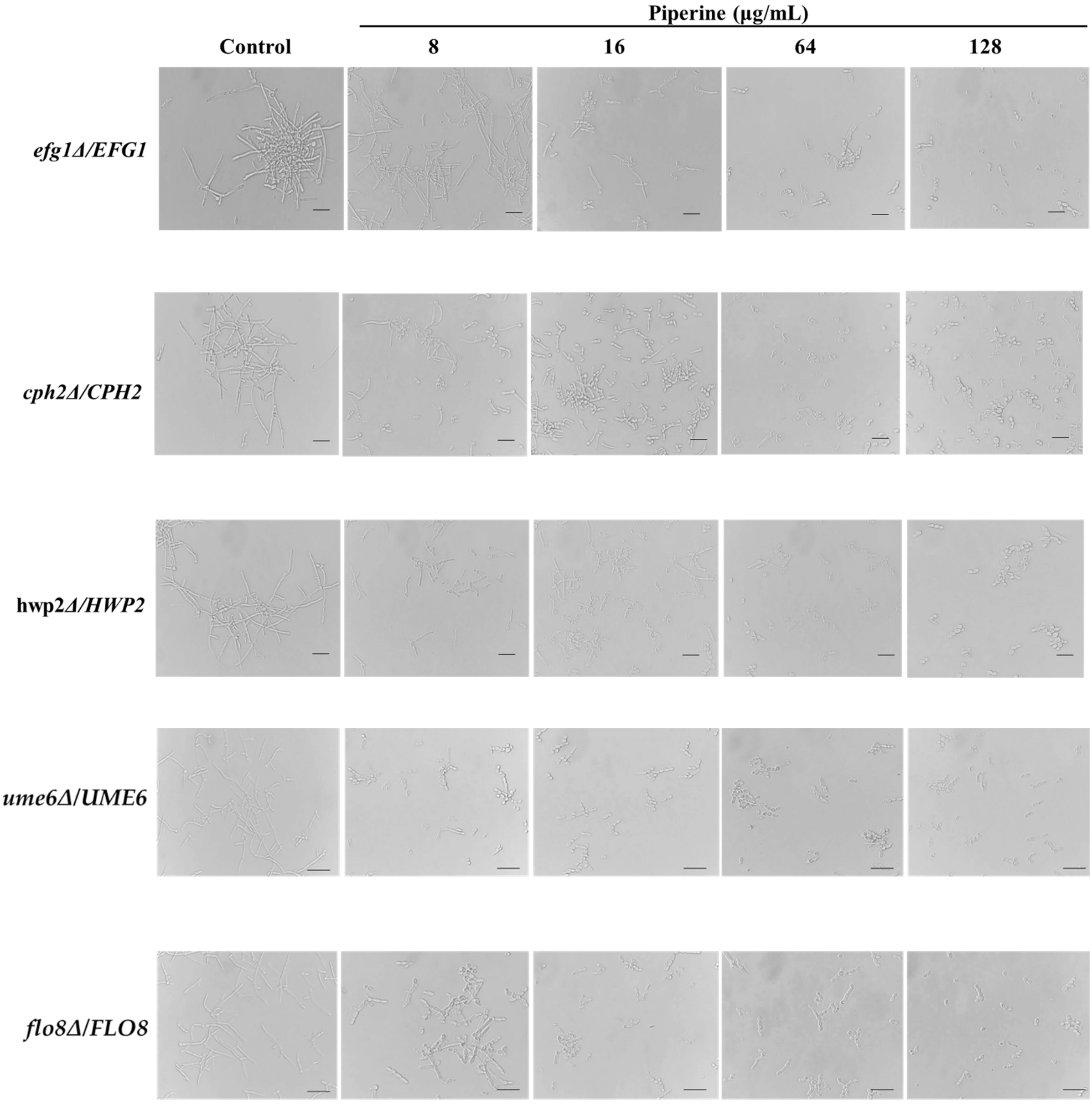

tup1Δ/TUP1

nrg1Δ/NRG1

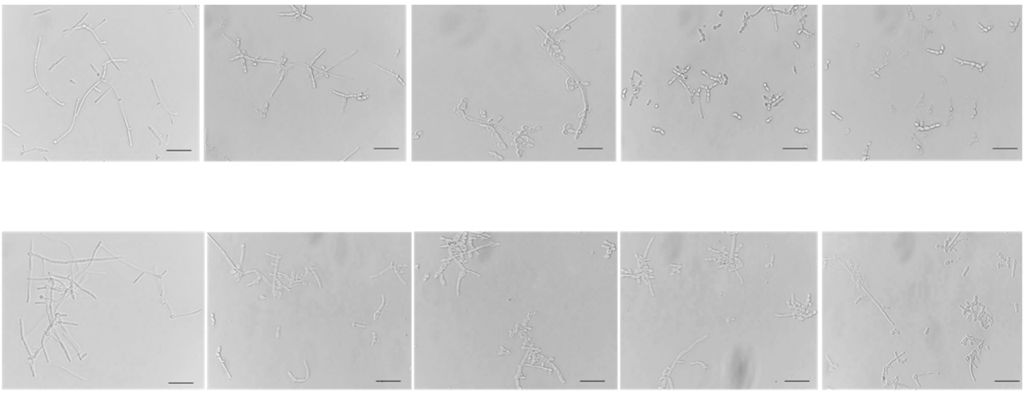

Figure S6. Piperine slight affects yeast to hyphal transition in liquid medium in mutant strains of *C. albicans*. Photographic record. The scale of the images represents 50 μm.
